# A random-object-kinematogram plugin for web-based research: implementing oriented objects enables varying coherence levels and stimulus congruency levels

**DOI:** 10.3758/s13428-021-01767-3

**Published:** 2022-05-03

**Authors:** Younes Strittmatter, Markus Wolfgang Hermann Spitzer, Andrea Kiesel

**Affiliations:** Department of Cognitive Psychology, University of Freiburg, Freiburg im Breisgau, Germany

**Keywords:** Random-dot kinematogram, JsPsych plugin, Online experiments, Object implementation, Congruency effect

## Abstract

One of the recent major advances in cognitive psychology research has been the option of web-based in addition to lab-based experimental research. This option fosters experimental research by increasing the pace and size of collecting data sets. Importantly, web-based research profits heavily from integrating tasks that are frequently applied in cognitive psychology into open access software. For instance, an open access random-dot kinematogram (RDK) plugin has recently been integrated into the jsPsych software for web-based research. This plugin allows researchers to implement experimental tasks with varying coherence levels (with that varying task difficulty) of moving dots or varying signal to noise ratios of colored dots. Here, we introduce the random-object kinematogram (ROK) plugin for the jsPsych software which, among other new features, enables researchers to include oriented objects (e.g., triangles or arrows) instead of dots as stimuli. This permits experiments with feature congruency (e.g., upwards-moving triangles pointing upwards) or incongruency (e.g., upwards-moving triangles pointing downwards), allowing to induce gradual degrees of stimulus interference, in addition to gradual degrees of task difficulty. We elaborate on possible set-ups with this plugin in two experiments examining participants’ RTs and error rates on different combinations of coherence and congruency levels. Results showed increased RTs and error rates on trials with lower coherence percentages, and on trials with lower congruency levels. We discuss other new features of the ROK plugin and conclude that the possibility of gradually varying the coherence level and congruency level independently from each other offers novel possibilities when conducting web-based experiments.

## Introduction

The number of online experiments conducted throughout the past decade has increased massively in several academic fields, such as cognitive psychology, developmental psychology, and social psychology, with web-based experiments revealing similar data quality, but faster data collection, as lab-based experiments (Crump et al., 2013; Buhrmester et al., 2011; Ramsey et al., 2016). A major milestone to foster web-based research was the introduction of open access software such as jsPych (de Leeuw, 2015) and the implementation of plugins for experimental set-ups that can easily be applied with this software. For instance, the random dot kinematogram (RDK) plugin (see Rajananda et al., 2018) allows researchers to easily apply RDK stimulus displays (Kayser et al., 2010; Britten et al., 1992; Shadlen et al., 1996; Purcell & Kiani, 2016; Marques et al., 2018; Guterstam et al., 2020; Guterstam & Graziano, 2020; von Lautz et al., 2019; Benetti et al., 2021; Kang et al., 2021; Shenhav et al., 2018; Spitzer et al., 2019; Spitzer et al., in press; Krueger et al., 2017; Mante et al., 2013; Danielmeier et al., 2011; Ritz & Shenhav, 2019) in order to implement the random-dot motion task in online experiments (for example see: Bhui, 2019; Musslick et al., 2019; Krzeminski & Zhang, 2021).

The RDK stimulus displays contain varying numbers of dots that move in a target, opposing to the target, or random direction and participants are instructed to detect in which specific direction the majority of dots move (i.e., the coherent direction). One feature of this task is that the difficulty of the task can be adjusted gradually by decreasing the motion coherence (to make the task harder) or increasing the motion coherence (to make the task easier). In addition to the motion identification task, dots may be presented in two different colors, enabling a color discrimination task. For this color task, participants are instructed to detect the dominant of two different colors while the ratio of colors can be varied to allow different task difficulties (Spitzer et al., 2019; Musslick et al., 2019; Shenhav et al., 2018; Krueger et al., 2017; Steyvers et al., 2019).

Here, we introduce an advanced version of the RDK plugin - the random object kinematogram (ROK) plugin. The ROK plugin incorporates the functionalities of the RDK-plugin but additionally enables researchers to (a) upload visual objects instead of dots as stimuli, (b) implement multiple ROK stimulus displays at the same time, and (c) integrate background pictures (see Table 1 for an overview of ROK plugin parameter settings). This set of new functionalities allows more flexibility on the stimulus presentation and thus expands the possibilities of experimental tasks one can design using the ROK plugin which we describe in the following sections. The ROK plugin is fully available and free to use on https://github.com/younesStrittmatter/ROK-plugin. A documentation as well as a demonstration of the ROK plugin is available on this GitHub link, but a short description of how to use the plugin shall briefly be described. The jsPsych JavaScript library (https://www.jspsych.org/) is a prerequisite to run the plugin. If jsPsych is installed, copy the file jspsych-rok.js (located in the plugins folder of the repository) into the plugins-folder of the jsPsych library. To use the stimuli provided with this plugin, copy the res-folder into your experiments project folder. See the documentary (docs) folder or the example folder for further instructions. You can use the examples folder (alongside the jsPsych library) as template to run your own experiments.

**Table 1:** Parameters and their description for the ROK plugin

Parameter	Description (ROK)
choices	The valid keys that the subject can press to indicate a response.
correct_choice	The correct keys for that trial.
trial_duration	The length of stimulus presentation. Zero for endless loop.
response_ends_trial	If true, then any valid key will end the trial.
number_of_apertures	Number of apertures. If greater then one, other parameters of trial should be arrays.
density_unit_area	If this parameter is set, number_of_objects is interpreted as number_of_objects per density_unit_area (in pixels*pixels)
number_of_oobs	The number of oriented objects (oobs) per set in the stimulus.
coherence	The proportion of dots that move together in the coherent direction. Range is 0 to 1.
opposite_coherence	The proportion of moving in the direction opposite of the coherent direction. Range is 0 to (1-coherence).
coherent_movement_direction	The direction of coherent motion in degrees (0 degree = right).
coherent_orientation	The orientation of the objects in degree (0 degree = right).
coherence_movement	The percentage of oriented objects moving in the coherent direction.
coherence_orientation	The percentage of objects that are oriented in the coherent orientation.
coherence_movement_opposite	The percentage of oriented objects moving in the direction opposite of the coherent direction.
coherence_orientation_opposite	The percentage of objects that are oriented opposite of the coherent orientation.
movement_speed	The movement speed of the oobs in (percentage of aperature_width)/second.
movement_speed_randomisation	The percentage of randomisation in movement speed; 0 = all orientated objects move with defined
	speed in movement_speed; 100 = movement speeds from 0 to 2x movement_speed.
random_movement_type	Type of random movement: 0 direction is random but fixed; 1 movement direction of incoherent oobs changes over time.
random_orientation_type	Type of random movement; 0 - orientation is random but fixed, 1 - orientation of incoherent oobs changes over time.
oob_size	The size of the oriented objects in percentage of aperture_width.
oob_color	The color of the oobs.
background_color	The color of the background.
background_image	Background image, can be.
aperture_width	The width of the aperture in pixels.
aperture_height	he height of the aperture in pixels.
aperture_position_left	Position of midpoint of aperture in x direction in percentage of window width (0 being left, 100 being right).
aperture_position_top	Position of midpoint of aperture in y direction in percentage of window width (0 being top, 100 being bottom).
aperture_shape	0 - rectangular, 1 - elliptic.
stimulus_type	Appearance of stimulus (0-triangles, 1-circle,2-square,3-bird, 4-image).
stimulus_image	Pictures of stimuli, can be key-framed (animated) or randomised, see documentation inside the plugin code.
stimulus_image_keyframes	Number of keyframes in stimulus images.
stimulus_keyframe_time	Time between keyframes in seconds.
stimulus_mirror	Mirror image instead of rotating (1 - x axis, 2 - y axis).
	Can be useful for oobs that have two orientation axis (e.g front to back and up and down).
prompt	Prompt that is presented above the stimulus.
fade_out	Fade the oobs on the edges of the aperture.
experiment_congruency_mode	Sets experiment to congruency mode, experiment_main_task has to be set (0 = movement or 1 = orientation)
	if this is set to 1 or 2. The congruency of the task does only apply to coherent oobs of the main task.
	If this is set to 1 the remaining oobs secondary feature (the non-task feature) is set at random.
	If this is set to 2 the remaining oobs have the same movement and orientation direction.
experiment_main_task	Sets the main task when experiment is in congruency mode. The congruency of the other task then only applies
	to non random oobs of main task (0 - movement task, 1 - orientation task).
units	Units in which size and speed of oobs is expressed (null - percentage of aperture width, px - pixels.

**Fig. 1:**
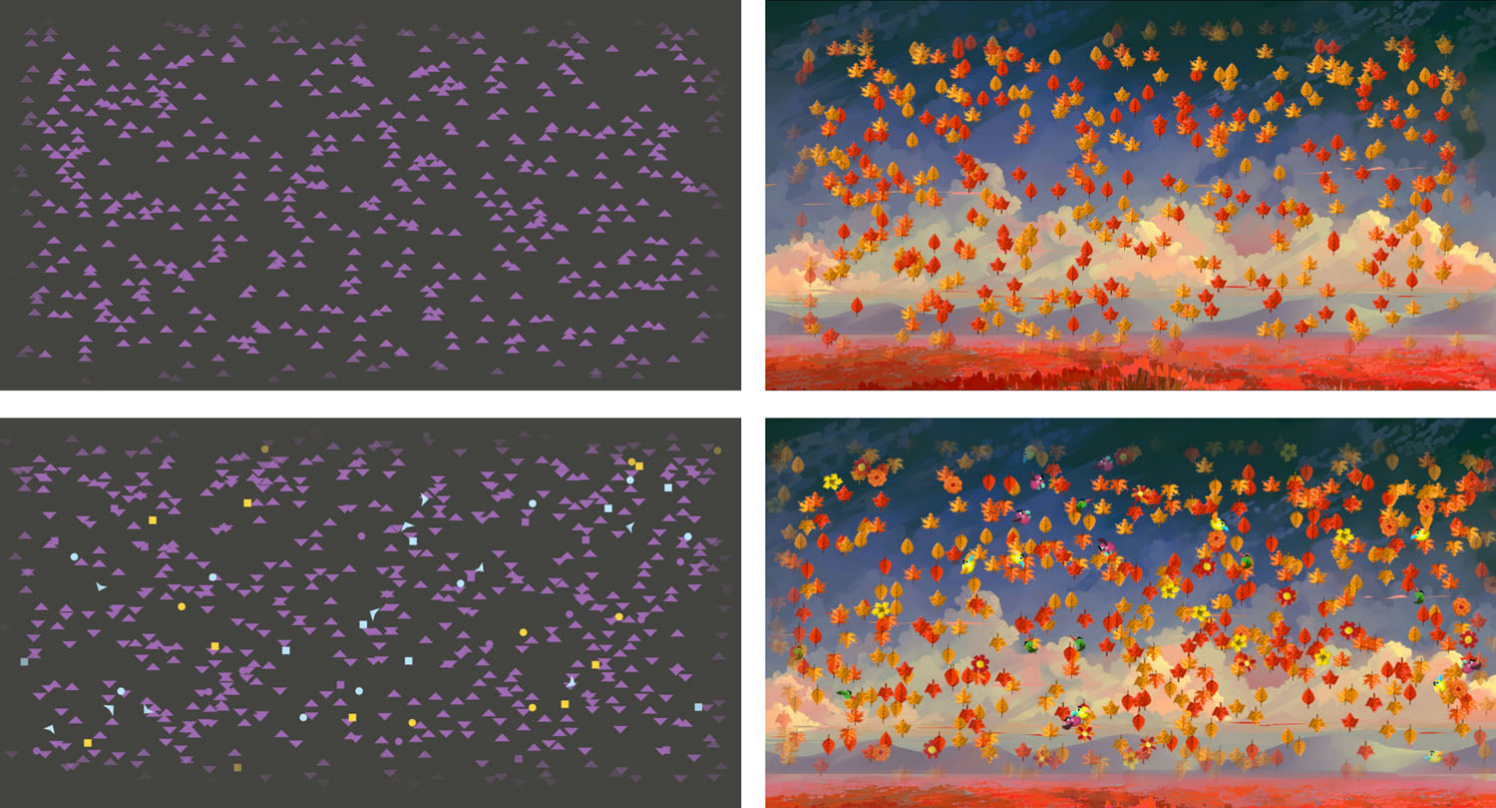
Experiment stimuli for Experiment 1 (left panel; oriented isosceles triangle objects) and Experiment 2 (right panel; oriented leaf objects). In both tasks, participants were instructed to respond to the coherent moving direction (up or down) or the coherent orientation direction (up or down). The *upper row* depicts the target stimulus layer, for each task respectively. The *bottom row* shows the target and distractor stimulus layer presented at the same time, for each task respectively

The major novelty of the ROK plugin is the implementation of object stimuli instead of dot stimuli. Object stimuli can include features which may lead to interference on the stimulus level. For example, imagine a set of arrow stimuli, instead of dot stimuli, presented with the ROK plugin (see Fig. 1). Moving arrows now expand the feature space of the ROK, with a proportion of these arrows *moving* in a coherent direction, while the remaining arrows move in random direction *and*, at the same time, a proportion of these arrows *orienting* in a coherent direction, while the remaining arrows are oriented in random direction. With this setup, one can instruct participants to respond to one of the two stimulus dimensions: moving direction or orientation direction. If objects move and are oriented in only two directions (e.g., up and down) and participants are instructed to detect the dominant movement direction, moving arrows can be oriented in the same direction, which we refer to as a congruency level of 100%, or be directed in opposing directions, which we refer to as a congruency level of 0%. However, any congruency level between 100% and 0% is possible. For example, the congruency level of the two stimuli can be 50%. In this case, if participants are instructed to respond to the motion task, with the two directions up and down, half of the objects would be oriented upwards, while the other half would be oriented downwards. In sum, with this setup of two stimulus dimensions (moving direction and orientation direction), the ROK plugin enables flexible research with a task of varying difficulty levels by changing the coherence of the instructed dimension, *and* varying congruency levels, by changing the coherence of the irrelevant dimension.

Another new feature of the ROK plugin is that researchers can present multiple layers of ROK stimuli at once in one trial (see Fig. 1) and the described features of coherence and congruency level can be manipulated for each stimulus layer, respectively. The objects of each layer can be presented “on top of each other”, in a defined order, or in randomized order. Here, we labeled the stimulus layer participants are instructed to respond to as the *target layer* and labeled the stimulus layer with distractor stimuli as *distractor layer*. For example, if participants are instructed to respond to upwards- or downwards-moving and oriented arrows, then the target layer would be the random-arrow kinematogram. If now a second stimulus layer (e.g., a random-square kinematogram) would simultaneously be presented but participants are instructed to disregard these stimuli, this layer would then be denoted as the distractor layer.

Finally, users may implement background pictures for their ROKs. This allows researchers to implement the ROK stimuli in different contexts, depending on the background picture.

### The present experiments

**Fig. 2:**
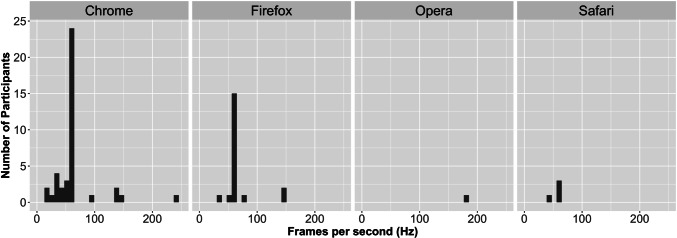
Data collection quality for different internet browser. The average frame per second (Hz) for each participant for both experiments was computed and plotted in a histogram for the browser the participant used. Most participants used Chrome or Firefox while only few participants used Opera or Safari

Here, we applied the ROK plugin for the jsPsych software in two experiments and demonstrated some of the possible usage variants. Therefore, we first built the ROK plugin (for an overview of the parameter settings of the ROK plugin, see Table 1; see Table 2 for an overview of which parameters additionally get generated) and tested the data collection quality (see Fig. 2). Please note that the ROK plugin interfaces with the jspsych-resize plugin to allow for standardized displays. We then conducted Experiment 1, which included isosceles triangles as stimuli objects and with two tasks, (1) detecting the majority of upwards- or downwards-moving triangles, or (2) identifying the majority of upwards- or downwards-oriented triangles. With these two tasks, we applied different coherence and congruency levels and asked whether lower coherence and congruency levels would increase RTs and error rates and tested whether the two factors (coherence and congruency level) interacted with each other (Analysis 1a & 1b). Moreover, we tested whether the effect of coherence and congruency level differed between tasks. Finally, we investigated whether the addition of a distraction layer would increase RTs and error rates compared to no distraction layer while we kept the coherence and congruency level constant (Analysis 1c & 1d). Each of these analyses was then replicated with leaves as stimuli objects and on a naturalistic background in Analysis 2a-d.

**Table 2:** The plugin collects all parameter data of Table 1 and the following data for each trial

Parameter	Description (ROK)
rt	The response time in ms for the subject to make a response.
key_press	The key that the subject pressed. The value corresponds to the JavaScript Char Code (Key Code).
correct	Whether or not the subject’s key press corresponded to those provided in correct_choice.
frame_rate	The average frame rate for the trial. 0 denotes that the subject responded before the appearance of the second frame.
number_of_frames	The number of frames that was shown in this trial.
frame_rate_array	The array that holds the number of milliseconds for each frame in this trial.
canvas_width	The width of the canvas in pixels.
canvas_hight	The height of the canvas in pixels.

In both experiments, we expected RTs and error rates to increase with decreasing coherence levels and with decreasing congruency levels. We expected increased RTs and error rates when a distractor layer is added compared to non-distractor trials. Finally, we had no prior hypothesis which of the two tasks (movement vs. orientation) was more difficult, in terms of faster or slower responses and higher or lower error rates on one of the two tasks.

## Experiment 1

Experiment 1 was conducted to examine whether the possibility to gradually adjust the coherence *and* the congruency level of the task affected RTs and error rates. We applied purple isosceles triangles moving and being oriented upwards or downwards as the target stimulus layer. Participants were instructed to either respond to the motion direction or the orientation direction of the triangles throughout ten blocks. On the first five blocks participants were instructed to respond to one of the two tasks while they were instructed to respond to the other task on the second half of the blocks. The task order was counterbalanced across participants. On block 2-5, participants responded to one of the two tasks, with all possible combinations (12 combinations) of three coherence levels (90%, 75%, and 60%) and four congruency levels (100%, 75%, 25%, and 0%). On blocks 7-10, participants responded to the other task and the same coherence (90%, 75%, and 60%) and congruency level (100%, 75%, 25%, and 0%) combinations. This change of task instruction on blocks 7-10 may have led to another level of interference, namely response interference, as participants learnt how to respond to the now irrelevant task in the first half of the experiment. We added this task order factor (labeled as *order*) in our analyses to investigate whether the interference effect differed between the first and second half of the experiment. In addition, we asked if the coherence and congruency level effect was different between the two tasks. More precisely, the first two analyses asked whether RTs (Analysis 1a) and error rates (Analysis 1b) depended on coherence, congruency level, order, and task, including all main effects and all possible interactions between these four factors.

On block 1 and block 6, we additionally collected data on different trials with fixed coherence and congruency levels but applied a second distractor stimulus layer. Specifically, the coherence level was set to 65% and the congruency level to 50%. In addition in 10% of the trials, a distractor stimulus layer was added. We asked whether RTs (Analysis 1c) and error rates (Analysis 1d) increased on trials which included a distractor stimulus layer compared to trials with no distractor stimulus layer, and whether this effect differed between tasks. Squares, points, and randomly oriented triangles, colored in turquoise or yellow, served as distractor stimuli layer, while the target layer was the same as in the other blocks.

Prior to any analyses, we examined the quality of the data collection by collecting the frame rate for each response. The ROK plugin automatically collects data of the average frame rate for a trial (see Table 2 for all parameters the ROK plugin collects in addition to the parameters described in Table 1). We plotted the average frames per second (Hz) of trial responses per participant and for each browser respectively in Figure 2. Please note that we merged the data for this plot from Experiments 1 and 2 to increase the number of participants per browser.

### Method

#### Participants

Thirty-four participants (13 females; 21 males; *M*_*age*_ = 27.73; *SD*_*age*_ = 5.42) were recruited with Prolific and paid £2.5 ($3.5) for their participation (max. 30 min). All participants provided informed consent before the start of the experiment and were told that they could stop the experiment at any given point. The sample size was based on a power analysis which expected a medium effect of the main effect of congruency level with an effect size of *d* = .5 and a power of 80% (Faul et al., 2007). Participants were only included in the final data analysis if they responded with an accuracy above 60%.

#### Stimuli

Stimuli were presented with the jsPsych software (de Leeuw, 2015). A total of 600 purple isosceles triangles presented on black background comprised a ROK stimulus display in this experiment (see Fig. 1 for a stimulus example). Objects moved and were oriented in the same two directions (up or down); yet, please note that any angle of direction could be set in the ROK plugin (for ROK plugin details see Table 1). The object size was set to 1% of aperture width and the motion speed set to 3% of aperture width per second.

Only in block 1 and block 6, a second layer which served as a distractor layer was implemented on 10% of the trials (drawn randomly). This distractor layer comprised 70 squares, points, or randomly oriented triangles colored in turquoise and yellow with an object size of 1% of aperture width and a motion speed of 3% of aperture width per second.

#### Procedure

Each trial included a fixation cross presented for 500 ms, followed by a ROK stimulus which was presented for up to 2000 ms. Participants were instructed to respond to the motion direction or the orientation direction of the ROK stimulus by responding with the key press ‘F’ and ‘J’. Half of the participants were instructed to press ‘F’ for mostly upwards-moving objects and mostly upwards-oriented objects, and ‘J’ for mostly downwards-moving objects and mostly downwards-oriented objects. The instructions reversed for the other half of participants.

At the beginning of the experiment, participants were provided with instructions and 16 training trials. Participants responded to a total of ten blocks each of which included 72 trials. Participants responded to one of the two tasks in blocks 1-5 and then responded to the other task on blocks 6-10. The task order was counterbalanced across participants.

During training trials and after accurate responses, the word ‘CORRECT’ colored in green ink was presented as feedback on the screen after each trial. After accurate responses in experimental trials no such feedback was given. After an incorrect response, the word ‘FALSE’ colored in red ink was presented as feedback on the screen after each trial (i.e., on training trials and on trials in blocks 1-10). When the participants did not respond within 2000 ms, the words ‘TOO SLOW’ appeared in red ink (on training trials and on trials in blocks 1-10).

Blocks 1 and 6 addressed a different research question and comprised different analyses than blocks 2-5 and 7-10. The differences between these blocks are described in detail in the following two sections.

The first and sixth block included the target stimulus layer of purple triangles and a distractor stimulus layer with points, squares and randomly oriented triangles colored in turquoise and yellow which appeared with a probability of 10% of the trials. On these two blocks the coherence was set to 65%, indicating that 65% of the objects moved (or oriented) upwards or downwards while 35% of the objects moved (or were oriented) in the opposite direction. The congruency level was set to 50%, indicating that the same amount of irrelevant stimuli moved (or were oriented) up and down.

On blocks 2-5 and blocks 7-10, only the target layer was used as ROK stimuli. The coherence of the task varied between the three levels 90%, 75%, and 60% and the congruency level varied between the four levels 100%, 75%, 25%, and 0%. Each of the 12 coherence-congruency level combinations was applied six times within a block, respectively, summing up to a total of 72 trials per block. The coherence indicated the proportion of upwards- and downwards-moving (or were oriented) objects. For instance, 90% coherence indicated that 90% of the objects moved (or were oriented) in the target direction, while 10% of the objects moved (or were oriented) towards the non-target direction. The congruency level expressed the proportion of congruent to incongruent objects with 100% indicating only congruent objects and 0% indicating only incongruent objects. For example, for the motion task, a congruency level of 100% indicated that all objects were oriented towards the target direction, while a congruency level of 0% indicated that all objects were oriented towards the opposite direction of the target direction. In other words, 0% congruency indicated 100% incongruency. For the orientation task, a congruency level of e.g., 75% indicated that 450 (of the 600) objects moved towards the target direction, while a congruency level of 25% indicated that 150 (of the 600) objects moved towards the opposite direction of the target direction Thus, the 25% congruency level expressed 75% incongruency.

#### Independent and dependent variables

We conducted four separate analyses. Two only included blocks 2-5 and blocks 7-10 and investigated the effect of coherence and congruency level on RTs (Analyses 1a) and error rates (Analyses 1b). Another two analyses only included blocks 1 and 6 and investigated the effect of distractor stimuli on RTs (Analyses 1c) and error rates (Analyses 1d). The first two analyses comprised a total of four independent variables (*coherence, congruency level, order* and *task*). A continuous coherence variable included three coherence levels (90%, 75%, and 60%), indicating the percentage of objects moving in the target direction. A continuous congruency level variable, included four congruency levels (100%, 75%, 25%, and 0%), indicating the percentage of objects of the irrelevant feature moving or orienting into the coherent task direction. A categorical order variable indicated whether responses were made in the first or second half of the experiment, to investigate whether the effect of having responded to the other task on the first half of the experiment, induced an additional response congruency effect, on top of the stimulus congruency effect, and thus increased the effect of congruency level on the second half of the experiment. A categorical task variable indicated the current task.

Analyses 1c and 1d comprised two independent variables only: a categorical task variable indicating the current target task and a categorical distractor variable indicating whether a distractor layer was absent or present. As described above, the coherence and congruency level were fixed to a specific value (65% coherence and 50% congruency level) on trials of block 1 and block 6 and thus, coherence and congruency level was not included in Analysis 1c and 1d. Dependent variables on all four analyses comprised participants’ RTs (Analyses 1a & c) and error rates (Analyses 1b & d) on stimulus responses, respectively.

#### Data analysis

The statistical analysis was conducted with R (R Core Team, 2013). Linear mixed models were conducted with the lmerTest package (Kuznetsova et al., 2017) and logistic mixed models were conducted with the lme4 package (Bates et al., 2014). Plots and tables were generated with the sjPlot package (Lüdecke, 2020). We used linear mixed models instead of analysis of variances (ANOVAs) since linear mixed models are less prone to inflated Type 1 errors (Quené & Van Den Bergh, 2004; Quené, 2008; Judd et al., 2017).

In Analysis 1a and 1b, we analyzed the blocks that did not include distractor trials (blocks 2-5 & blocks 7-10) and investigated RTs and error rates as a function of coherence, congruency level, order, and task, including all main effects and interaction effects. We expected slower responses as well as higher error rates on lower coherence levels and on more incongruent, compared to more congruent, trials. In addition, we asked whether response congruency further interacted with stimulus congruency, leading to a larger congruency effect in trials with stimulus congruency and response congruency (second half) compared to trials where only stimulus congruency was manipulated (first half). We had no expectations on the effect of task and on the interactions between the four variables.

Random effects were selected based on the least complex random effect structure which accounted for most of the random effect variance (see Bates et al., 2015). In detail, we carried out a principal component analysis on the random effects with all main effects as random slopes and a random intercept for participants. We then assessed whether the number of random effects addressed all principal components, or whether too many random effects (random slopes in specific) were included in the model. If principal components explained close to zero variance (< 0.001), we excluded random slope effects explaining the least variance and fitted the model again. We followed this procedure until each principal component of the random effect structure explained 0.001 or more variance. Importantly, fixed effect results were only examined for the finally selected model. We report which of the random slope effects were included in the winning model. Please note that a random intercept for participants was always included in the model to account for individual differences on the dependent variable.

In Analysis 1c and d, we analyzed the first and sixth block of the experiment to investigate RTs and error rates as a function of distraction and task. We expected slower RTs and higher error rates on trials which included a distractor layer compared to trials that did not include distractors. Here, random effects comprised a random intercept for each participant. No random slopes were included since the model did not account for more variance when including random slopes (Bates et al., 2015).

### Results

We report the results of all main effects and all significant interactions. The complete results of each analysis is provided in tables.

#### Exclusion criteria

The following exclusion criteria were applied. We excluded all trials with an RT below 200 ms and with no responses within 2000 ms (2.77%). For RT analyses, all incorrect responses and all responses following incorrect responses (38.70%) were excluded. Finally, two participants were excluded prior to the data analysis due to an accuracy rate below 60%.

#### Analysis 1a: RT as a function of coherence, congruency level, order, and task

**Fig. 3:**
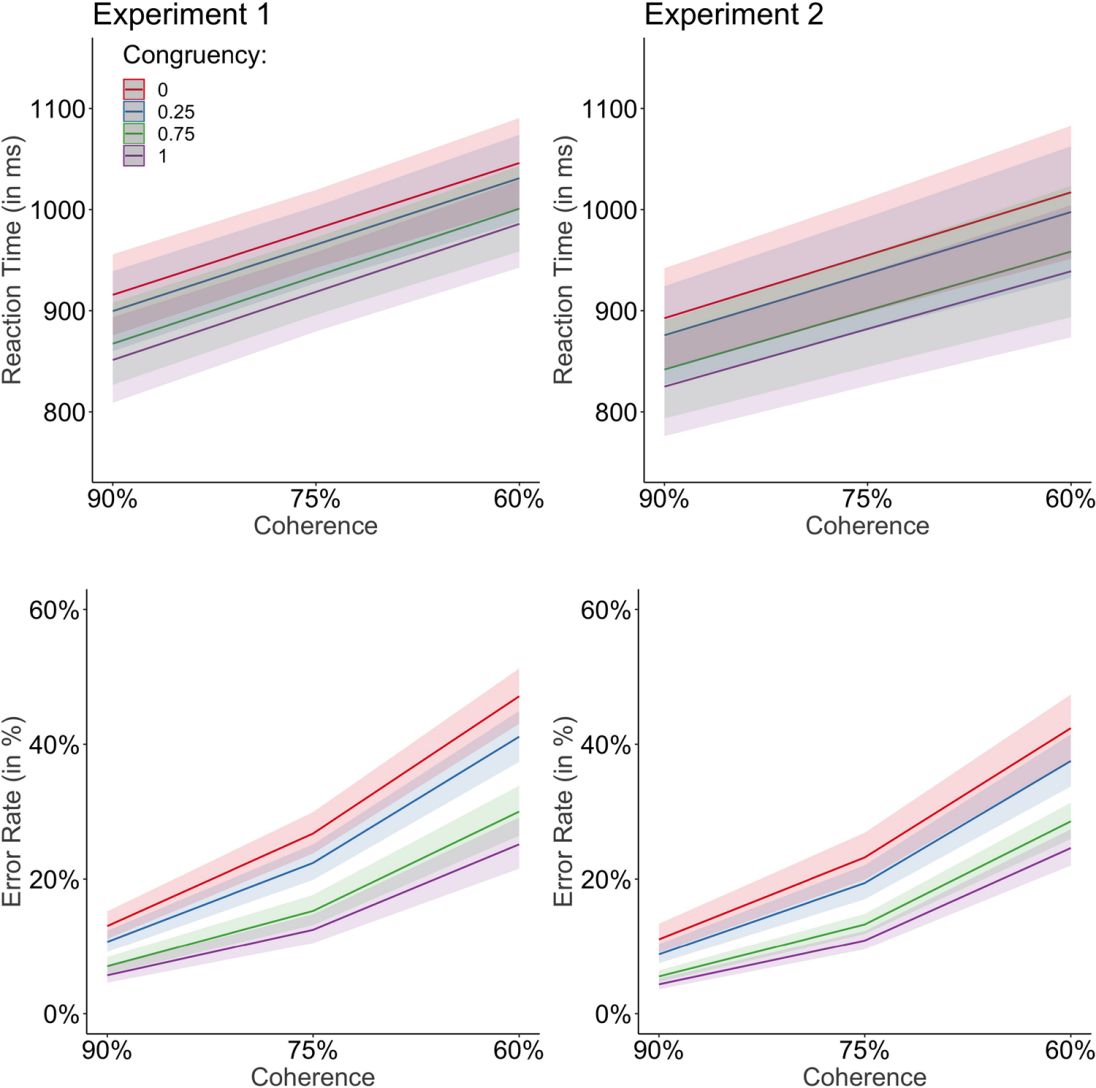
RTs and error rates as a function of coherence and congruency level for each experiment, respectively. RTs and error rates increased with decreasing coherence percentages and lower congruency levels. The results of Experiment 2 mimick the results of Experiment 1

RTs as a function of coherence and congruency level are depicted in Fig. 3. All regression results are listed in Table 3. Random effects included the variables coherence, congruency level, and task, as a random slope to account for individual differences on these three variables. The order term was not included as the model did not account for more variance when including order as a random slope and we sought to apply the least complex model according to Bates et al., (2015).

**Table 3:** Estimates (Betas), 95% Confidence Intervals, t-Values, and p-Values of the Linear Mixed Model of Analysis 1a (Experiment 1) and 2a (Experiment 2)

	Exp 1: RT					Exp 2: RT		
Coeffcient	Estimates	Conf. Int (95%)	t-Value	p-Value	Estimates	Conf. Int (95%)	t-Value	p-Value
Intercept	1305.90	1201.89 - 1409.91	24.61	<**0.001**	1266.90	1150.35 - 1383.44	21.31	<**0.001**
Coherence	-433.55	-554.51 - -312.59	-7.02	<**0.001**	-412.13	-508.09 - -316.17	-8.42	<**0.001**
Congruency	-51.06	-130.96 - 28.84	-1.26	0.210	-95.55	-166.70 - -24.40	-2.63	**0.008**
Order	-47.92	-107.50 - 11.66	-1.58	0.115	2.20	-53.36 - 57.75	0.08	0.938
Task	85.45	25.88 - 145.03	2.81	0.005	21.31	-34.24 - 76.86	0.75	0.452
Coherence:Congruency	-15.17	-116.58 - 86.23	-0.29	0.769	30.07	-60.74 - 120.88	0.65	0.516
Coherence:Order	51.11	-16.02 - 118.24	1.49	0.136	-29.96	-89.62 - 29.70	-0.98	0.325
Congruency:Order	39.52	-39.38 - 118.42	0.98	0.326	5.76	-64.74 - 76.27	0.16	0.873
Coherence:Task	-135.42	-202.55 - -68.29	-3.95	< 0.001	51.98	-7.68 - 111.63	1.71	0.088
Congruency:Task	89.10	10.20 - 168.00	2.21	0.027	60.39	-10.11 - 130.90	1.68	0.093
Order:Task	-55.91	-159.92 - 48.10	-1.05	0.292	-65.64	-182.19 - 50.90	-1.10	0.270
Coherence:Congruency:Order	-76.68	-178.05 - 24.69	-1.48	0.138	-21.40	-112.17 - 69.38	-0.46	0.644
Coherence:Congruency:Task	-81.69	-183.06 - 19.67	-1.58	0.114	-81.03	-171.80 - 9.75	-1.75	0.080
Coherence:Order:Task	50.20	-70.77 - 171.16	0.81	0.416	66.42	-29.54 - 162.38	1.36	0.175
Congruency:Order:Task	-58.43	-138.33 - 21.47	-1.43	0.152	50.41	-20.74 - 121.56	1.39	0.165
Coherence:Congruency:Order:Task	82.89	-18.52 - 184.30	1.60	0.109	-75.14	-165.95 - 15.66	-1.62	0.105
Random Effects								
*σ*^2^	66721.10				59390.03			
*τ*_00_	$61955.67_{subject\_id}$				$94580.53_{subject\_id}$			
*τ*_11_	$77678.06_{subject\_id.coherence}$				$46192.15_{subject\_id.coherence}$			
	$1106.42_{subject\_id.congruency}$				$677.44_{subject\_id.congruency}$			
	$6123.98_{subject\_id.task1}$				$7636.41_{subject\_id.task1}$			
*ρ*_01_	-0.90				-0.94			
	-0.37				-0.05			
	0.24				0.13			
Observations	11172				12363			

Sigma squared denotes the residual variance of the random effects. Tau00 indicates the variance between participants. Tau 11 indicates the variance between participants on random slopes for each random effect. Rho indicates the correlations between participants’ random intercept and their random slope, respectively

The main effect of coherence was significant (*b* = − 433.96; *t* = − 7.22; *p* < .001), with faster responses on higher coherence levels. The main effect of congruency level was not significant (*b* = -51.26; *t* = -1.26; *p* = .208). The main effect of order was not significant (*b* = -47.86; *t* = -1.59; *p* = .113). The main effect of task was significant (*b* = 85.49; *t* = 2.83; *p* = .005), with slower responses on the orientation task compared to the motion task. The interaction between coherence and task was significant (*b*= − 135.43; *t*= − 3.96; *p*<.001), with a steeper increase in RTs with lower coherence levels on the orientation task compared to the motion task. The interaction between congruency level and task was significant (*b* = 88.95; *t* = 2.21; *p* = .027), with an increased congruency effect for the motion task compared to the orientation task. None of the other interactions were significant.

#### Analysis 1b: Error rates as a function of coherence, congruency level, order, and task

Error rates as a function of coherence and congruency level are depicted in Fig. 3. All error rate results are listed in Table 4. Random effects included the variables congruency level, and task, as a random slope to account for individual differences on these two variables. This was the least complex random effect structure to account for most of the variance of the model.

**Table 4:** Log-Odds (Betas), 95% Confidence Intervals, t-Values, and p-Values of the Logistic Mixed Model of Analysis 1b (Experiment 1) and 2b (Experiment 2)

	Exp 1: Error Rate				Exp 2: Error Rate			
Coeffcient	Log-Odds	Conf. Int (95%)	*z*-Value	*p*-value	Log-Odds	Conf. Int (95%)	*z*-Value	*p*-Value
Intercept	3.45	3.07− 3.83	17.67	<**0.001**	3.25	2.84 − 3.66	15.64	<**0.001**
Coherence	− 5.95	− 6.44 − 5.45	− 23.72	<**0.001**	− 5.93	− 6.44 − 5.42	− 22.92	<**0.001**
Congruency	− 1.11	− 1.73 − 0.49	− 3.50	<**0.001**	− 0.43	− 1.09 − 0.23	− 1.29	0.198
Order	0.10	− 0.27 − 0.46	0.52	0.603	− 0.10	− 0.47 − 0.28	− 0.51	0.613
Task	0.32	− 0.05 − 0.69	1.72	0.085	− 0.27	− 0.64 − 0.11	− 1.39	0.164
Coherence:Congruency	0.23	− 0.63 − 1.08	0.52	0.602	− 0.64	− 1.54 − 0.27	− 1.38	0.168
Coherence:Order	− 0.03	− 0.52 − 0.46	− 0.10	0.917	0.24	− 0.26 − 0.75	0.95	0.343
Congruency:Order	− 0.36	− 0.97 − 0.25	− 1.16	0.248	0.35	− 0.29 − 0.98	1.07	0.284
Order:Task	− 0.34	− 0.73 − 0.04	− 1.76	0.079	0.18	− 0.22 − 0.59	0.88	0.377
Coherence:Congruency:Order	0.36	− 0.49 − 1.21	0.83	0.406	− 0.67	− 1.57 − 0.24	− 1.45	0.147
Coherence:Congruency:Task	− 0.40	− 1.26 − 0.45	− 0.93	0.352	0.02	− 0.88 − 0.92	0.05	0.962
Coherence:Order:Task	0.51	0.02 − 1.00	2.03	**0.042**	− 0.34	− 0.85 − 0.17	− 1.31	0.190
Congruency:Order:Task	0.24	− 0.38 − 0.86	0.77	0.444	− 0.91	− 1.56 − 0.25	− 2.71	**0.007**
Coherence:Congruency:Order:Task	− 0.19	− 1.04 − 0.66	− 0.44	0.662	1.58	0.68 − 2.48	3.43	**0.001**
Random Effects								
*σ*^2^	3.29				3.29			
*τ*_00_	$0.17_{\text {subject\_id}}$				$0.29_{\text {subject\_id}}$			
*τ*_11_	$0.13_{\text {subject\_id.coherence}}$				$0.24_{\text {subject\_id.coherence}}$			
	$0.08_{\text {subject\_id.congruency}}$				$0.06_{\text {subject\_id.congruency}}$			
*ρ*_01_	− 0.10				− 0.79			
	− 0.71				− 0.65			
Observations	18225				18801			

Sigma squared denotes the residual variance of the random effects. Tau00 indicates the variance between participants. Tau 11 indicates the variance between participants on random slopes for each random effect. Rho indicates the correlations between participants’ random intercept and their random slope, respectively

The main effect of coherence was significant (*b*= − 5.95; *z*= − 23.72; *p*<.001), with less erroneous responses on higher coherence levels. The main effect of congruency level was significant (*b*= − 1.11; *z*= − 3.50; *p*<.001), with more erroneous responses on trials with higher incongruency levels. The main effect of order was not significant (*b* = 0.10; *z* = 0.52; *p* = .603). The main effect of task was not significant (*b* = 0.32; *z* = 1.72; *p* = .085). The interaction of coherence and task was significant (*b* = -0.60; *z* = -2.41; *p* = .016), with a steeper increase in error rates with lower coherence levels on the orientation task compared to the motion task. The interaction between coherence, order, and task was significant (*b* = 0.51; *z* = 2.03; *p* = .042), because the increase in error rates on lower coherence levels was steeper for the orientation task than the motion task on the first half of the experiment, but steeper for the motion task than the orientation task in the second half of the experiment. Please note, however, that the robustness of this three-way interaction has to be interpreted with caution, as our sample size was rather small.

#### Analysis 1c: RTs as a function of distraction

RTs as a function of distractor layer and task are shown in Table 5. The main effect of distraction was significant (*b*= − 94.74; *t*= − 8.48; *p*<.001), with faster responses on non-distractor trials compared to distractor trials. The main effect of task was significant (*b*= − 23.34; *t*= − 2.07; *p*=.039), with slower responses on the orientation task than the motion task. The interaction between distractor and task was significant (*b*= 39.00; *t*= 3.49; *p*<.001), with slower RTs on the motion task than the orientation task on distractor trials, but faster responses on the motion task than the orientation task on no distractor trials.

**Table 5:** Estimates (Betas), 95% Confidence Intervals, t-Values, and p-Values of the Linear Mixed Model of Analysis 1c (Experiment 1) and 2c (Experiment 2)

	Exp 1: RT				Exp 2: RT			
Coeffcient	Estimates	Conf. Int (95%)	*t*-Value	*p*-Value	Estimates	Conf. Int (95%)	*t*-Value	*p*-Value
Intercept	1226.03	1177.48 − 1274.58	49.50	<**0.001**	1176.11	1096.17 ?1256.06	28.83	<**0.001**
Distractor	− 94.74	− 116.65 − 72.84	− 8.48	<**0.001**	− 71.38	− 93.10 − 49.66	− 6.44	<**0.001**
Task	− 23.34	− 45.47 − 1.21	− 2.07	**0.039**	16.40	− 5.43 − 38.23	1.47	0.141
Distractor:Task	39.00	17.08 − 60.92	3.49	<**0.001**	6.87	− 14.87 − 28.62	0.62	0.535
Random Effects								
*σ*^2^	80177.57				77353.51			
*τ*_00_	$15564.72_{\text {subject\_id}}$				$52276.72_{\text {subject\_id}}$			
Observations	1946				2028			

Sigma squared denotes the residual variance of the random effects. Tau00 indicates the variance between participants

#### Analysis 1d: Error rates as a function of distraction

Error rates as a function of distractor layer and task are shown in Table 6. The main effect of distraction was not significant (*b* = -0.07; *z* = -1.24; *p* = .215). The main effect of task was significant (*b* = -0.11; *z* = -2.08; *p* = .038) with more errors on the motion task compared to the orientation task. The interaction of distractor and task was significant (*b* = 0.13; *z* = 2.47; *p* = .014) with more errors on the motion task, compared to the orientation task, on distractor trials, but less errors on the motion task, compared to the orientation task, on non-distractor trials.

**Table 6:** Log-Odds (Betas), 95% Confidence Intervals, t-Values, and p-Values of the Logistic Mixed Model of Analysis 1d (Experiment 1) and 2d (Experiment 2)

	Exp 1: Error Rate				Exp 2: Error Rate			
Coeffcient	Log-Odds	Conf. Int (95%)	*z*-Value	*p*-Value	Log-Odds	Conf. Int (95%)	*z*-Value	*p*-Value
Intercept	− 0.55	− 0.70 − 0.41	− 7.54	<**0.001**	− 0.54	− 0.69 − 0.40	− 7.40	<**0.001**
Distractor	− 0.07	− 0.17 − 0.04	− 1.24	0.215	− 0.08	− 0.18 − 0.02	− 1.60	0.110
Task	− 0.11	− 0.21 − 0.01	− 2.08	**0.038**	0.04	− 0.06 − 0.14	0.83	0.404
Distractor:Task	0.13	0.03 − 0.23	2.47	**0.014**	− 0.02	− 0.12 − 0.08	− 0.40	0.687
Random Effects								
*σ*^2^	3.29				3.29			
*τ*_00_	$0.08_{\text {subject\_id}}$				$0.09_{\text {subject\_id}}$			
Observations	4507				4732			

Sigma squared denotes the residual variance of the random effects. Tau00 indicates the variance between participants

### Discussion

The purpose of Experiment 1 was to apply two of the new features of the ROK plugin for the jsPsych software: (a) implementing objects instead of dots and (b) applying two ROK layers at the same time. To address the possibility of implementing objects instead of dots to enable varying degrees of stimulus interference, we investigated the effect of different combinations of coherence *and* congruency levels on RTs and error rates. Results revealed that RTs and error rates depended on the coherence and congruency level. With respect to the main effect of coherence, the results revealed slower and more erroneous responses on lower coherence levels. These results suggest that participants’ evidence accumulation took more time and was more error-prone on trials with lower signal to noise ratio levels. These results were in line with our predictions and with previous observations (e.g., Purcell and Kiani, 2016; Spitzer et al., 2019; Shenhav et al., 2018). Regarding the main effect of congruency level, participants revealed a significant effect of congruency level on error rates with more errors on trials with increasingly incongruent stimuli. Please note that the RT result on congruency level was not significant, but the descriptive pattern of this result was in line with the error rate result and does not speak in favor of a speed-accuracy trade-off (see Fig. 3). To the best of our knowledge, these coherence and congruency level results are the first to show that different degrees of stimulus interference on the random-object motion task effected participants’ performance significantly over and beyond the effect of different coherence levels.

We further investigated whether the task order influenced participants RTs or error rates. The motivation behind this analysis was that participants might have learnt on how to respond to the first task on blocks 1-5 and that this learnt response mapping induced a response congruency effect during the second half of the experiment (namely blocks 6-10). In this case, the effect of congruency level would be larger on the second half of the experiment compared to the first half of the experiment. However, the interaction of congruency level and order was not significant and thus did not provide evidence for an additional effect of response congruency on top of the stimulus congruency effect in the second half of the experiment. But please note that the absence of proof is not proof of absence.

In addition to these effects, we asked whether the effect of coherence, congruency level, and order further differed between the two tasks and whether one of the two tasks was easier than the other. Results revealed that the orientation task was more difficult than the motion task, as indicated by longer RTs and higher error rates on the orientation task than the motion task. The coherence variable interacted with task, showing a steeper increase in RT and error rates with lower coherence levels on the orientation task than the motion task. In addition, we observed a significant interaction of congruency and task for RTs (but not for error rates), revealing an increased congruency effect for the motion task than the orientation task. Interestingly, the coherence and congruency variables did not interact with each other. Again, the absence of this interaction does not provide evidence whether the effects of these two variables influenced participants orthogonally. However, the descriptive pattern of these results does not indicate an interaction effect of these two variables.

In two other separate analyses, we investigated whether the implementation of a distractor stimulus layer would effect RTs and error rates. Results supported our hypothesis with slower responses on distractor trials compared to non-distractor trials. This effect was not significant in error rates, but the result pattern descriptively points in the same direction. Besides, participants were slower and made more errors on the orientation task than the motion task. However, this main effect was driven by an increased difference between the two tasks on distractor trials.

In sum, Experiment 1 tested two new features of the ROK plugin: implementing objects instead of dots and applying two ROK layers at the same time. The implementation of objects instead of dots enables the integration of another feature, which, in this experiment, induced different degrees of stimulus congruency affecting participants’ performance. Finally, results provide evidence that applying a distractor stimulus layer in addition to the target stimulus layer increased participants’ RTs and error rates. In the following experiment, we asked whether the result pattern observed in Experiment 1 could be replicated with an experiment using more aesthetic stimuli and a background picture.

## Experiment 2

Experiment 2 was conducted to investigate whether results from Experiment 1 would replicate when objects changed to leaves instead of triangles and with a naturalistic background. The procedure of this experiment was the same as the one of Experiment 1, except for the change in objects and the inclusion of a background (see Fig. 1 for a ROK stimulus layer example).

### Method

#### Participants

As in Experiment 1, 34 participants (13 females; 21 males; *M*_*age*_ = 29.29; *SD*_*age*_ = 6.73) were recruited with Prolific and paid £2.5 ($3.5 ) for their participation (30 min). All participants agreed with the informed consent provided before the start of the experiment and were told that they could stop the experiment at any given time. The sample size was based on the same criteria as in Experiment 1. Participants were only included in the final data analysis if they responded with an accuracy above 60%.

#### Stimuli, procedure, variables and data analysis

Despite the change in stimuli and the addition of a background, the procedure, independent and dependent variables, and statistical analyses were the same as in Experiment 1. The random effect structure was kept the same in each analysis as in Experiment 1. All principal component analyses revealed that the random effect structure was not too complex with the proportion of variance explained by each principal component larger than zero.

### Results

We report all main effects and significant interactions in the text below. The results of each individual analysis are listed in tables.

#### Exclusion criteria

The following exclusion criteria were applied. We excluded all trials with an RT below 200 ms and with no responses within 2000 ms (1.77%). For RT analyses, all incorrect responses and all responses following incorrect responses (36.08%) were excluded. Finally, one participant was excluded prior to the data analysis due to an accuracy rate below 60%.

#### Analysis 2a: RTs as a function of coherence, congruency, order and task

RTs as a function of coherence and congruency level are depicted in Fig. 3. All regression results are listed in Table 3. The random effect structure was the same as in Experiment 1.

As in Experiment 1, the main effect of coherence was significant (*b*= − 412.69; *t*= − 8.62; *p*<.001), with slower responses on lower coherence levels. The main effect of congruency level was significant (*b* = -95.76; *t* = -2.64; *p* = .008), with slower responses on trials with higher incongruency levels. The main effect of order was not significant (*b* = 2.17; *t* = 0.08; *p* = .938). The main effect of task was not significant (*b* = -21.19; *t* = 0.75; *p* = .451). None of the interactions were significant.

#### Analysis 2b: Error rates as a function of coherence, congruency level, order, and task.

Error rates as a function of coherence and congruency level are depicted in Fig. 3. All regression results are listed in Table 4. The random effect structure was the same as in Experiment 1.

The main effect of coherence was significant (*b*= − 5.93; *z*= − 22.92; *p*<.001), with more erroneous responses on lower coherence levels. The main effect of congruency was not significant (*b* = -0.43; *z* = -1.29; *p* = .198), but the described result pattern was in the direction of a congruency effect. The main effect of order was not significant (*b* = -0.10; *z* = -0.51; *p* = .613). The main effect of task was not significant (*b* = -0.26; *z* = -1.38; *p* = .168). The three-way interaction of congruency, order, and task was significant (*b* = -0.91; *z* = -2.71; *p* = .007), with a stronger congruency effect for the orientation task in the first half of the experiment but a stronger congruency effect for the motion task in the second half of the experiment. Finally, the four-way interaction of coherence, congruency, task, and order was significant (*b* = 1.58; *z* = 3.43; *p* = .001) and was further explored in a post hoc analysis (see below). None of the remaining interactions were significant.

The significant four-way interaction was further explored with two separate analyses which investigated the effect of coherence, congruency level, and order for each task (motion and orientation) separately. The same regression model was therefore fitted for each task, respectively and without the task variable as a fixed effect and random slope effect. Results of the motion task model revealed a significant main effect for coherence (*b*= − 6.53; *z*= − 16.88; *p*<.001), with more erroneous responses on lower coherence levels. In addition, the results revealed a significant interaction between congruency and order (*b* = 1.20; *z* = 2.41; *p* = .015), with an increased congruency effect on the second half of the experiment. Finally, results showed a significant three-way interaction (*b* = -2.18; *z* = -3.12; *p* = .001), with a congruency effect on the first half of the experiment only for lower coherence levels and a congruency effect on the second half of the experiment for all coherence levels. Results of the orientation task model revealed a significant main effect for coherence (*b*= − 5.48; *z*= − 15.65; *p*<.001), with more erroneous responses on lower coherence levels, but no other significant main effects or significant interactions.

#### Analysis 2c: RTs as a function of distraction

The results of this analysis are listed in Table 5. The main effect of distraction was significant (*b* = -71.38; *t* = -6.44; *p* = .001), with slower responses on distractor trials compared to non-distractor trials. There was no main effect of task (*b* = 16.40; *t* = 1.47; *p* = .141) and no interaction between task and distractor (*b* = 6.87; *t* = 0.62; *p* = .535).

#### Analysis 2d: Error rates as a function of distraction

The results of this analysis are listed in Table 6. The main effect of distraction was not significant (*b* = -0.08; *z* = -1.60; *p* = .110) but the descriptive pattern of the results pointed towards more errors on distractor trials. There was no main effect of task (*b* = 0.04; *z* = 0.83; *p* = .404) and no interaction between task and distractor (*b* = -0.02; *z* = -0.40; *p* = .687).

### Discussion

The purpose of Experiment 2 was to replicate the result pattern of Experiment 1 with a different stimulus type, i.e., oriented leaves moving and orienting up or down on a naturalistic background. In line with Experiment 1, responses were slower and associated with higher error rates on lower coherence levels. In addition, participants were slower on incongruent compared to congruent trials and made marginally more errors on incongruent than congruent trials. A post hoc analysis on a significant four-way interaction suggested that the congruency effect increased on the second half of the experiment for the motion task, indicated by higher error rates on more incongruent trials than on more congruent trials. However, please note that this four-way interaction has to be interpreted with caution, as our sample size was rather small, and the effect was not observed in the first experiment. Future studies may investigate the robustness of this interaction effect. The two tasks did not significantly differ with respect to RTs and error rates in Experiment 2. As in the previous experiment, distractor trials increased RTs and error rates were marginally increased on distractor trials. Together, these results mimic the result pattern of Experiment 1 and thus, we conclude that the results of Experiment 1 are independent of the object style and the background.

## General discussion

In this research project, we developed and examined a ROK plugin for the jsPych software (de Leeuw, 2015). This ROK plugin has three major new features: (a) implementing objects instead of dots; (b) implementing several ROK layers on top of each other; and (c) implementing background pictures. We evaluated the quality of the data collection with two experiments (see Fig. 2), which additionally tested experimental setups using the ROK plugin. In detail, we implemented oriented objects as stimuli so that participants can be instructed to respond according to the motion (motion detection task with two directions ‘up’ and ‘down’) or the orientation (orientation detection task with two directions ‘up’ and ‘down’) of the majority of the objects. With this experimental setup, the coherence of the target feature could be varied. In addition, the amount of overlapping or opposing feature directions could be varied, enabling different degrees of congruency. We applied different combinations of coherence and congruency levels and investigated the influence on participants’ RTs and error rates with two experiments. The results of both experiments showed that lower coherence percentages increased participants’ RTs and error rates. In addition, lower congruency levels (that is more incongruent stimuli) either increased participants’ error rates (Experiment 1) or increased participants’ RTs (Experiment 2), while the other performance measure (RTs in Experiment 1 and error rates in Experiment 2) revealed the same descriptive, but non-significant, pattern. Finally, we provide evidence that the implementation of an additional distractor stimulus layer led to increased RTs and error rates, compared to trials with no additional distractor layer. In the remainder of the discussion, we further discuss each of the three new features in more detail.

### Implementing oriented objects

Implementing oriented objects instead of dots enlarges the experimental possibilities of the random-object motion task. First, a second stimulus dimension enables researchers to investigate stimulus interference effects by varying congruency levels in addition to coherence levels. This integration of continuously varying stimulus interference allows researchers to apply this ‘random-object motion task’ in a similar way to other tasks applied in cognitive psychology designed to induce stimulus interference. Thus, the setting is similar to, for example, the Stroop task, on which participants are instructed to respond to the ink color of color words, with either overlapping (congruent stimuli; e.g., RED) color word meaning and color ink or non-overlapping color word meaning and color ink (incongruent stimuli; e.g., RED) (Stroop, 1935; Cohen et al., 1990). Or it is similar to the flanker task, on which participants are instructed to respond to the direction of an arrow in the middle of a screen surrounded by arrows pointing in the same or opposing direction (Eriksen & Eriksen, 1974). Yet, in contrast to these two traditional tasks, the difficulty of the random-object motion task as well as the congruency level can be varied *continuously* expanding the possibilities of task usage considerably.

### Implementing several ROK stimulus layers at the same time

Multiple ROKs may be presented at the same time, allowing the possibility to implement distraction stimuli of another irrelevant stimulus dimension. Results provided evidence that participants’ performance decreased on trials with these additional distractor stimulus layers. This new feature may be especially relevant for experimental designs which need to induce distractor or even surprising stimuli (e.g. see Wessel, 2018; Wessel & Aron, 2017).

### Changing object visuals and backgrounds

Results of Experiment 2, in which more naturalistic visuals and a background were applied, revealed similar result patterns with regards to main effects of coherence and congruency level. Changing the aesthetic look, and with that the context of the stimulus layer of experiments, may be especially relevant for studies with younger cohorts, as children may understand the instructions better when responding to more aesthetic visuals such as upwards or downwards floating leaves in nature, as compared to upwards or downwards moving triangles on a black background. In addition, the option to change the background allows for an easy manipulation of contexts. This enables, for example, to consider context-specific adaptation to congruency levels (Braem et al., 2019); similar to the context-specific proportion congruency effect (Crump et al., 2008; Schmidt & Lemercier, 2019; Wendt & Kiesel, 2011; Crump et al., 2006; Heinemann et al., 2009) or context-specific retrieval effects if target stimulus features, response features or other task parameters repeat or switch context-specifically (Frings et al., 2020; Dignath et al., 2021; Dignath et al., 2019).

### Conclusions

In sum, this ROK plugin serves as a platform for various research experiments in the fields of cognitive psychology or even developmental psychology, with gradual degrees of task difficulty *and* task interference, with the possibility to apply distractors, and with the possibility to implement different contexts. We hope that other researchers may benefit from this broad feature space integrated in the ROK plugin when designing experiments.
